# Targeting Drug-Sensitive and -Resistant Strains of *Mycobacterium tuberculosis* by Inhibition of Src Family Kinases Lowers Disease Burden and Pathology

**DOI:** 10.1128/mSphere.00043-15

**Published:** 2016-04-13

**Authors:** Pallavi Chandra, R. S. Rajmani, Garima Verma, Neel Sarovar Bhavesh, Dhiraj Kumar

**Affiliations:** a Cellular Immunology Group, International Centre for Genetic Engineering and Biotechnology, New Delhi, India; b Transcriptional Regulation Group, International Centre for Genetic Engineering and Biotechnology, New Delhi, India; Washington University in St. Louis School of Medicine

**Keywords:** adjunct therapy, host-directed therapy, MDR-TB, XDR-TB

## Abstract

In view of emerging drug resistance among bacterial pathogens, including *Mycobacterium tuberculosis*, the development of novel therapeutic strategies is increasingly being sought. A recent paradigm in antituberculosis (anti-TB) drug development is to target the host molecules that are crucial for intracellular survival of the pathogen. We previously showed the importance of Src tyrosine kinases in mycobacterial pathogenesis. Here, we report that inhibition of Src significantly reduced survival of H37Rv as well as multidrug-resistant (MDR) and extremely drug-resistant (XDR) strains of *M. tuberculosis* in THP-1 macrophages. Src inhibition was also effective in controlling *M. tuberculosis* infection in guinea pigs. In guinea pigs, reduced *M. tuberculosis* burden due to Src inhibition also led to a marked decline in the disease pathology. In agreement with the theoretical framework of host-directed approaches against the pathogen, Src inhibition was equally effective against an XDR strain in controlling infection in guinea pigs. We propose that Src inhibitors could be developed into effective host-directed anti-TB drugs, which could be indiscriminately used against both drug-sensitive and drug-resistant strains of *M. tuberculosis*.

**IMPORTANCE** The existing treatment regimen for tuberculosis (TB) suffers from deficiencies like high doses of antibiotics, long treatment duration, and inability to kill persistent populations in an efficient manner. Together, these contribute to the emergence of drug-resistant tuberculosis. Recently, several host factors were identified which help intracellular survival of *Mycobacterium tuberculosis* within the macrophage. These factors serve as attractive targets for developing alternate therapeutic strategies against *M. tuberculosis*. This strategy promises to be effective against drug-resistant strains. The approach also has potential to considerably lower the risk of emergence of new drug-resistant strains. We explored tyrosine kinase Src as a host factor exploited by virulent *M. tuberculosis* for intracellular survival. We show that Src inhibition can effectively control tuberculosis in infected guinea pigs. Moreover, Src inhibition ameliorated TB-associated pathology in guinea pigs. Thus, Src inhibitors have strong potential to be developed as possible anti-TB drugs.

## Abstract (precis)

The existing treatment regimen for tuberculosis (TB) suffers from deficiencies like high doses of antibiotics, long treatment duration, and inability to kill persistent populations in an efficient manner. Together, these contribute to the emergence of drug-resistant tuberculosis. Recently, several host factors were identified which help intracellular survival of *Mycobacterium tuberculosis* within the macrophage. These factors serve as attractive targets for developing alternate therapeutic strategies against *M. tuberculosis*. This strategy promises to be effective against drug-resistant strains. The approach also has potential to considerably lower the risk of emergence of new drug-resistant strains. We explored tyrosine kinase Src as a host factor exploited by virulent *M. tuberculosis* for intracellular survival. We show that Src inhibition can effectively control tuberculosis in infected guinea pigs. Moreover, Src inhibition ameliorated TB-associated pathology in guinea pigs. Thus, Src inhibitors have strong potential to be developed as possible anti-TB drugs.

## INTRODUCTION

*Mycobacterium tuberculosis* is the causative agent of tuberculosis (TB), which is one of the leading infectious diseases in the world. According to the World Health Organization (WHO), the year 2013 saw 9 million new TB cases and 1.5 million TB deaths worldwide. The current approach for TB treatment is to target vital enzymes or processes of the pathogen through antibiotics that effectively kill them, like isoniazid, ethambutol, rifampin, etc. The antibiotic treatment typically requires nearly 6 to 9 months in order to completely clear the infection. In the past decade, however, the emergence of multidrug-resistant (MDR) and extensively drug-resistant (XDR) strains of *M. tuberculosis* has compounded the problems with tuberculosis treatment (1). Globally, 3.5% of new TB cases and 20.5% of previously treated cases are estimated to have MDR-TB. A significant drawback of antibiotics is that they exert tremendous selective pressure on the bacteria, which could lead to spontaneous mutations and drug resistance. Therefore, any new antibiotic developed through the classical approach risks running into newer drug-resistant strains, which may evolve through the selective constraint.

Host-directed approaches to target pathogens have evolved as an alternate approach for antimicrobial drug discovery. The underlying principle of such approaches is that the pathogen coopts host factors to help its intracellular survival. Therefore, by identifying and targeting these factors/processes, one can limit the ability of the pathogen to evade killing by the host (2). Interestingly, a large body of research implicates the coopting of host tyrosine kinases by intracellular pathogens. Bacterial pathogens such as *Pseudomonas aeruginosa*, *Salmonella enterica* serovar Typhimurium, *Shigella flexneri*, and *Helicobacter pylori* employ host tyrosine kinase Abl for entry and infection (3–6). Some viruses, like poxviruses, require Src and Abl family kinases for actin tail formation and subsequent virion release (7). Thus, in principle, host tyrosine kinases represent attractive targets for the control of intracellular pathogens. Indeed, studies have demonstrated the use of Gleevec, an inhibitor of Abl family tyrosine kinases, to drastically compromise bacterial as well as viral pathogen survival within hosts (4, 7, 8).

Targeting of host tyrosine kinases has also gained momentum in the field of anti-TB drug development (9, 10). The importance of Abl and related tyrosine kinases in mycobacterial infection was successfully established in studies using imatinib, an inhibitor of Abl tyrosine kinases (11). It was reported that inhibition of Abl tyrosine kinase resulted in phagosomal acidification, thereby enhancing *M. tuberculosis* killing (12). Earlier, we also reported that the tyrosine kinase Src regulated two vital antimycobacterial processes of phagosomal maturation, acidification and autophagy (13).

In this study, we report the targeting of host Src tyrosine kinases using the inhibitor AZD0530 to achieve *M. tuberculosis* killing. AZD0530 is a potent dual Src/Abl inhibitor, which was originally tested as an anticancer drug. It blocks the ATP binding site of Src kinases, thereby inhibiting subsequent activation (14, 15). Upon treatment with AZD0530 at doses which selectively inhibited only Src, we observed a significant reduction in the bacterial survival in THP-1 macrophages infected with H37Rv as well as drug-resistant clinical isolates of *M. tuberculosis*. Furthermore, testing the drug in infected guinea pigs revealed that animals on chemotherapy not only had reduced pathogen load but also had dramatically reduced necrotic granulomas. Thus, Src inhibitors are attractive candidates for inclusion in anti-TB treatment regimens.

## RESULTS

### Src inhibition affects intracellular growth of drug-sensitive and drug-resistant strains of *Mycobacterium tuberculosis* in THP-1 macrophages.

To determine the ideal drug concentration for Src inhibition in THP-1 macrophages, we monitored phospho-Src (Y416) levels in the host upon treatment with a range of AZD0530 doses. Western blotting showed that Src inhibition was best achieved at 20 µM and 40 µM concentrations (see Fig. S1A at http://www.icgeb.res.in/dksup/Supplemental_Information_Revised.pdf). Since AZD0530 is an Src/Abl dual kinase inhibitor, we also probed for the levels of phospho-c-Abl (Y245) to check the effect of AZD0530 treatment on the activity of this kinase (see Fig. S1A). Western blots did not show any inhibition in c-Abl activity; rather, there was a marginal increase in c-Abl phosphorylation at higher doses (see Fig. S1A). We confirmed by one-dimensional (1D) ^1^H nuclear magnetic resonance (NMR) spectroscopy that the compound AZD0530 used in this study showed >98% purity, as also claimed by the supplier (see Fig. S1B).

To assess the effect of AZD0530 treatment on intracellular survival of *M. tuberculosis*, phorbol myristate acetate (PMA)-differentiated THP-1 macrophages were infected with H37Rv at a multiplicity of infection (MOI) of 1:10. The culture medium was supplemented with AZD0530 at 0 h postinfection (see Materials and Methods) at three different doses of 10, 20, and 40 µM (see Fig. S2A at http://www.icgeb.res.in/dksup/Supplemental_Information_Revised.pdf). Intracellular H37Rv killing was assessed by comparing bacterial CFU counts in drug-treated samples to counts in untreated samples at 48, 72, and 96 h postinfection. In parallel, THP-1 macrophage viability was assessed for the samples by MTT [3-(4,5-dimethyl-2-thiazolyl)-2,5-diphenyl-2H-tetrazolium bromide] assay (see Fig. S2B). At a 10 µM dose, H37Rv killing was observed after 72 h of treatment, while at both 20 and 40 µM, we observed significant decline in H37Rv intracellular survival at 48 h (see Fig. S2A). Notably, at the 40 µM dose, we also observed significantly higher rates of death of THP-1 macrophages (see Fig. S2B). Based on these results, we selected a 20 µM concentration of AZD0530 for the *in vitro* experiments. We also checked, at any of these concentrations, that there was no decline in H37Rv survival in the broth culture, thus confirming that AZD0530 has no direct antibacterial effect (see Fig. S3A at http://www.icgeb.res.in/dksup/Supplemental_Information_Revised.pdf).

**FIG 1:**
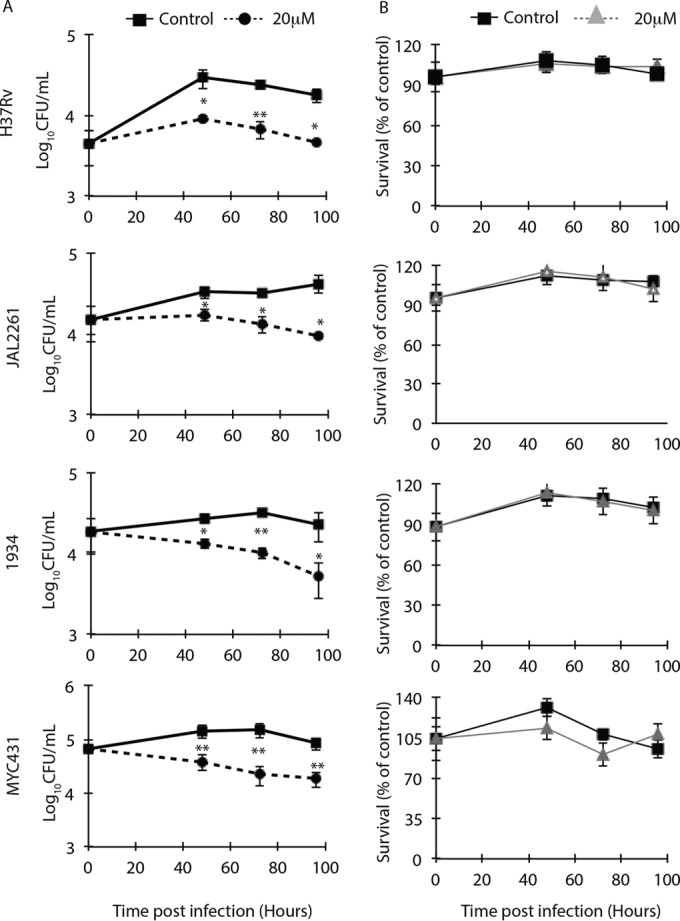
Effect of Src inhibition on intracellular survival of drug-sensitive and drug-resistant strains of *Mycobacterium tuberculosis* in THP-1 macrophages. (A) PMA-differentiated THP-1 macrophages infected with *M. tuberculosis* strains as described in the text were treated with AZD0530 at a 20 µM concentration. Cell lysates were plated on 7H11 agar plates at 48, 72, and 96 h postinfection. (B) Host cell viability under each of these conditions was assessed using MTT assay. (Values are averages ± standard deviations; *, *P* < 0.01; **, *P* < 0.001.)

Having established the dose, we extended our study to monitor the effect of Src inhibition on drug-resistant clinical isolates of *M. tuberculosis*, namely, JAL2261 and 1934 (multidrug-resistant isolates) and MYC431 (extremely drug-resistant isolate) (Table 1) in addition to the laboratory strain H37Rv (Fig. 1A). All three drug-resistant *M. tuberculosis* strains were not found to be susceptible to AZD0530 in the broth culture (see Fig. S3B, C, and D). PMA-differentiated THP-1 macrophages were infected with the above *M. tuberculosis* strains at an MOI of 1:10, and drug treatment was performed at a 20 µM concentration for 48, 72, and 96 h. In all these cases, AZD0530 was able to significantly reduce bacterial load in the infected cells. The intracellular bacillary load of H37Rv showed 60 to 90% reduction across all the time points (Fig. 1A). The intracellular bacillary load of MDR strains JAL2261 and 1934 was reduced by 50 to 80% with AZD0530 treatment (Fig. 1A). In the case of XDR strain MYC431, the bacillary load was reduced by 75 to 80% with treatment (Fig. 1A). There was no adverse effect on cellular survival upon treatment of MDR or XDR strain-infected macrophages with AZD0530 (Fig. 1B).

**TABLE 1:** Isolates used in this study^a^

Isolate	Resistance to drug:				Clade
	S	I	R	E	
H37Rv	−	−	−	−	T
JAL2261	−	+	+	+	Manu
1934	+	+	−	+	Manu
MYC431	+	+	+	+	Beijing

a Abbreviations and symbols: +, resistance; −, susceptibility; S, streptomycin; I, isoniazid; R, rifampin; E, ethambutol.

### Src inhibition promotes phagosomal acidification and xenophagy flux in macrophages.

**FIG 2:**
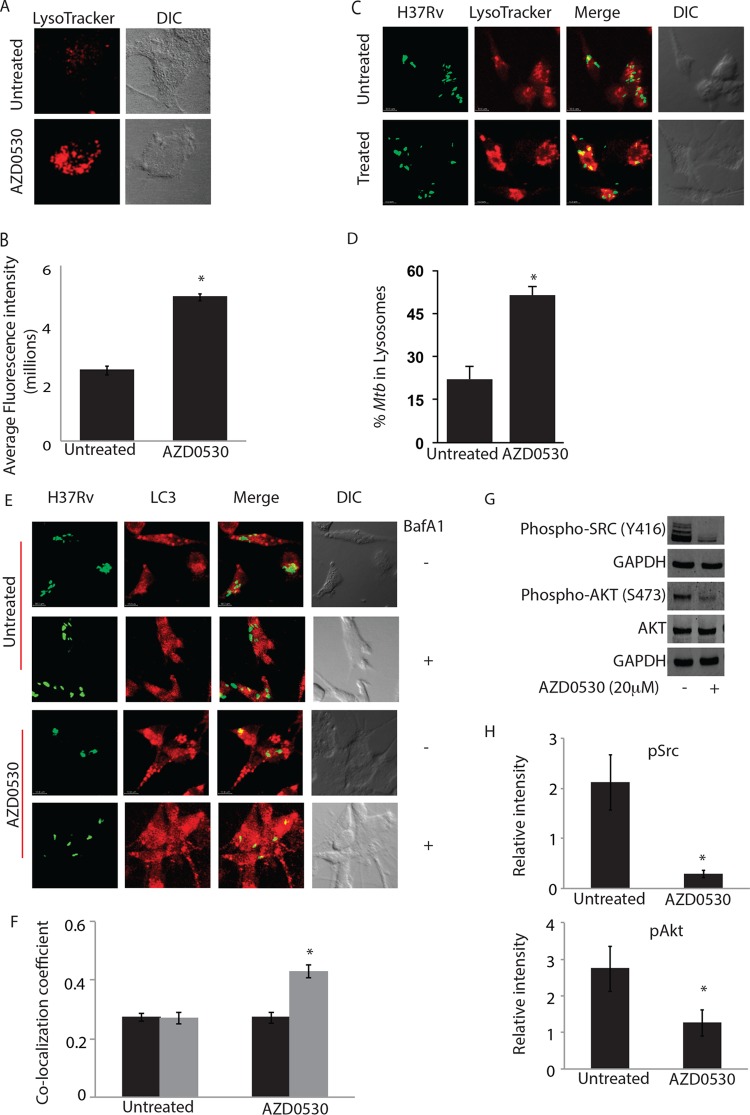
Src inhibition promotes phagosome acidification and autophagy in *Mycobacterium tuberculosis*-infected macrophages. (A) Uninfected THP-1 cells were treated with 20 µM AZD0530 for 48 h. The number of acidified lysosomes (LysoTracker, red) drastically increased upon treatment. DIC, differential interference contrast. (B) Average LysoTracker intensity per cell was calculated in uninfected untreated or AZD0530-treated THP-1 macrophages. The plot represents data from more than 100 cells across three different fields from two different experiments (values ± standard errors of the means; *, *P* value < 0.05). (C) H37Rv-infected THP-1 cells were treated with 20 µM AZD0530 for 48 h. Confocal images clearly show an increase in colocalization (merge, yellow regions) of *M. tuberculosis* (green) and acidified lysosomes (red) upon treatment. (D) Percentage of H37Rv colocalizing with acidified lysosomes upon AZD0530 treatment (20 µM, 48 h) is depicted as bar plots (average ± standard deviation; *, *P* value < 0.001). (E) PKH67-labeled H37Rv-infected THP-1 cells were left untreated or treated with 20 µM AZD0530 for 48 h. For the final 3 h, cells from both sets were either treated with BafA1 or left untreated to assess autophagy flux. Cells were fixed and stained using anti-LC3 antibody. (F) Colocalization coefficient (M1) of H37Rv with LC3 in AZD0530-treated and untreated sets in the presence or absence of BafA1 was calculated. The plot represents data from more than 100 cells across three fields from two different experiments (values ± standard errors of the means; *, *P* value < 0.01). (G) Western blots showing levels of indicated proteins in H37Rv-infected THP-1 cells, with or without AZD0530 treatment (20 µM), after 48 h of infection. GAPDH, glyceraldehyde-3-phosphate dehydrogenase. (H) Western blot intensities were estimated using Odyssey software. Values were normalized to the loading control and plotted as relative intensity of uninfected and untreated control set (values ± standard errors of the means; *, *P* value < 0.05).

Classically, phagosomal acidification has been described as a major mechanism employed by host macrophages for *M. tuberculosis* killing (16). We had previously shown that upon treatment with PP2, a peptide inhibitor of Src, more *M. tuberculosis* bacilli localized to the acidified lysosomes (13). We infected THP-1 macrophages with PKH67-labeled bacteria and visualized acidified lysosomes in the cells using LysoTracker dye. Microscopy analysis revealed intense LysoTracker staining in the cells, both uninfected and infected, that were treated with AZD0530 (Fig. 2A to C). Furthermore, we observed a significant increase, about 2-fold, in the percentage of H37Rv colocalizing with the acidified lysosomes upon AZD0530 treatment (Fig. 2D).

A key mechanism of host defense in addition to phagosomal acidification is autophagy. The role of autophagy in the regulation of intracellular survival of *M. tuberculosis* has been well described (17). In a recent study, we showed that H37Rv selectively blocks maturation of those autophagosomes where it resides, i.e., xenophagosomes (18). We therefore checked whether AZD0530 treatment could result in the maturation of xenophagosomes (Fig. 2E). Colocalization of H37Rv with LC3 increased in cells that were treated with AZD0530 in the presence of BafA1 with respect to the control cells (Fig. 2F). This reflected an increase in the rate of maturation of H37Rv-containing autophagosomes in AZD0530-treated cells. No such effect of BafA1 treatment could be observed in cells that were not treated with AZD0530 (Fig. 2F). We also monitored the effect of AZD0530 treatment on phosphorylation of Akt, which regulates autophagy through mToR (19, 20). AZD0530 treatment resulted in decreased phosphorylation of Src and AKT in H37Rv-infected THP-1 macrophages (Fig. 2G and H). The effect of AZD0530 on AKT phosphorylation was not infection specific, as even in the uninfected cells, AZD0530 treatment resulted in a decrease in AKT phosphorylation (see Fig. S4 at http://www.icgeb.res.in/dksup/Supplemental_Information_Revised.pdf).

### AZD0530 treatment reduces infection in guinea pigs infected with *Mycobacterium tuberculosis*.

**FIG 3:**
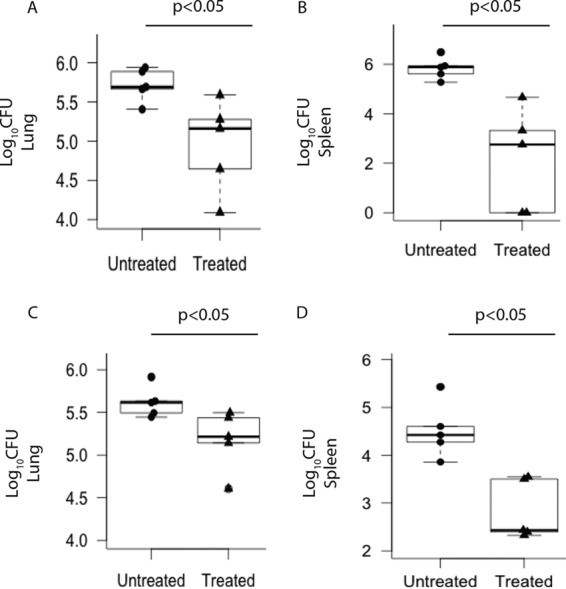
AZD0530 reduces infection in guinea pigs infected with *M. tuberculosis*. Outbred Dunkin-Hartley guinea pigs (*n* = 5) were infected with *M. tuberculosis* and were administered AZD0530 at a dose of 10 mg/kg. The bacillary load was determined by plating lung and spleen homogenates of treated and untreated animals. Bacterial CFU in whole lung and spleen tissues are shown here as box plots. (A and B) H37Rv CFU obtained at 8 weeks posttreatment in whole lung and whole spleen, respectively. (C and D) CFU from whole lung and whole spleen, respectively, in MYC431-infected guinea pigs at 8 weeks posttreatment. Each point represents data obtained from a single animal (*P* value < 0.05); *P* values were determined using the Mann-Whitney U rank sum test.

After we had checked the potential of AZD0530 *in vitro*, the drug was then tested in the guinea pig model of TB. Outbred Dunkin-Hartley guinea pigs were infected with H37Rv via the pulmonary route in a Wisconsin-Madison aerosol chamber. Nearly 150 bacilli were lodged in the guinea pig lungs as monitored by plating the lung homogenates 1 day after the aerosol challenge. Pathogen colonization was confirmed 15 days later by plating lung and spleen tissue homogenates from four infected animals. AZD0530 was administered to the animals by oral gavage 15 days post-aerosol challenge, at a dose of 10 mg/kg of body weight every alternate day. We started the treatment at day 15 because it was reported that at a similar load of infection in guinea pigs, *M. tuberculosis* grows exponentially until the first 2 weeks before stagnating (21). The dose of 10 mg/kg was calculated as a human-equivalent dose of 125 mg, i.e., ~2 mg/kg, used in other clinical studies (22). We are aware that the calculation of human-equivalent doses is mostly in the context of toxicity and may not be applicable for biological effects; nonetheless, in the absence of any other reference we thought it could be the best strategy to calculate equivalent doses for the guinea pigs. The effect of treatment in the infected animals (*n* = 5) was estimated by plating the lung and spleen homogenates after 8 weeks of treatment. Plots in Fig. 3 compare numbers of viable bacilli in whole lung of treated guinea pigs to those in the untreated ones. AZD0530 treatment significantly reduced *M. tuberculosis* infection in the lungs at 8 weeks postinfection (Fig. 3A). Furthermore, analysis of bacterial CFU obtained from spleen homogenates revealed a significant reduction in the hematogenous spread of infection. The plot in Fig. 3B clearly shows that the treatment reduced the bacterial load of the infected animals in the spleen by 8-fold at 8 weeks. Thus, AZD0530 drug therapy not only controlled infection at the primary site, i.e., lungs, but also minimized its systemic spread.

In addition, the efficacy of Src inhibition in reducing pathogen loads of MYC431 (XDR-TB) was tested in guinea pigs. Guinea pigs infected with MYC431 (*n* = 5) were administered AZD0530 at 10 mg/kg for a period of 8 weeks. Here, too, AZD0530 treatment resulted in a significant decrease in bacterial CFU in the lung and spleen homogenates compared to the untreated animals (Fig. 3C and D). One noticeable observation in this experiment was that in the H37Rv-infected guinea pigs, at week 8, CFU from the spleen were almost comparable with the lung CFU (Fig. 3). However, in MYC431-infected animals, lung CFU were significantly higher than the CFU from spleen (Fig. 3).

### AZD0530 reduces gross pathological damage in guinea pigs infected with *Mycobacterium tuberculosis*.

**FIG 4:**
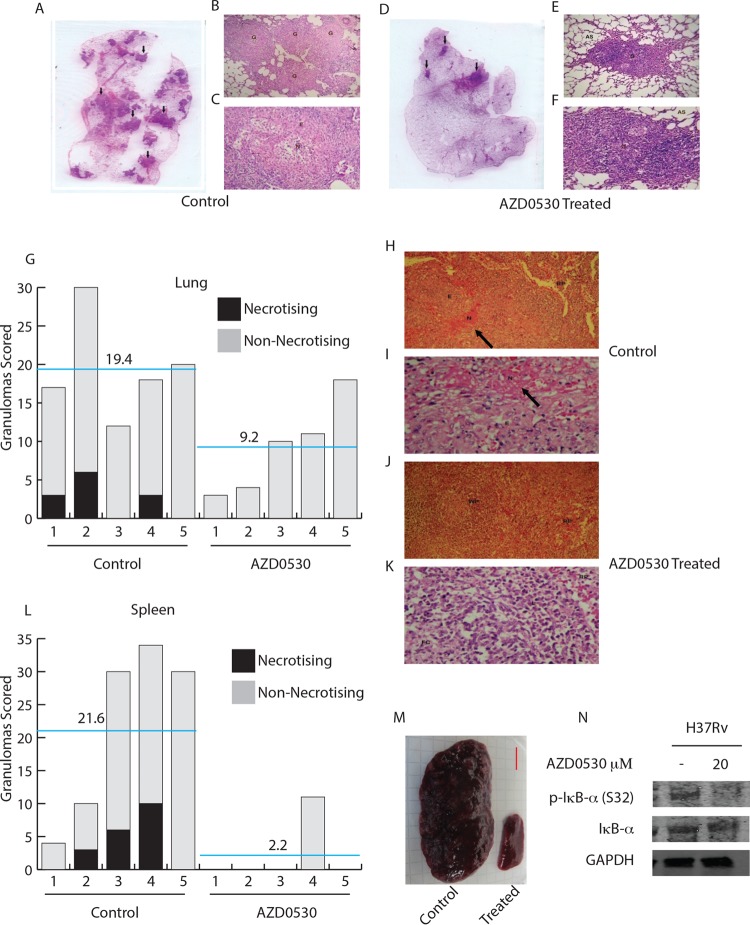
Reduced pathological damage in lungs and spleens of guinea pigs infected with H37Rv upon AZD0530 treatment. After 8 weeks of treatment, the lung and spleen tissues of the test animals were harvested and subjected to histopathological analyses. (A) Lung tissue from an untreated animal with granulomatous lesions (black arrows) is shown here. (B) Low-power photomicrograph (×40) of this lung tissue shows multiple granulomas in the lung parenchyma. (C) High-power photomicrograph (×100) showing the cellular composition in one of the granulomas. A central zone of necrosis surrounded by epithelioid cells can be observed. (D) The lung tissue from a treated animal showing lesser granulomatous lesions (black arrows). (E and F) No necrotic zones were observed in the granulomas at ×40 (E) and ×100 (F) magnifications. (G) Plot representing number of granulomas (necrotizing and nonnecrotizing) counted in lung sections from treated and untreated animals. The blue lines represent the average value for the untreated or treated sets of animals. (H) A section of spleen from an untreated animal at ×100 magnification is shown here. A large granuloma with central necrosis in the white pulp of the spleen can be observed (N). (I) High-power photomicrograph (×400) from the granuloma shows necrosis (N) in the upper half of the image with epithelioid cells in the lower half. (J) Spleens from the treated animals had no granulomatous lesions. A section of spleen from a treated animal showing normal splenic parenchyma is presented here at ×100 magnification. (K) High-power photomicrograph (×400) of the same section shows a portion of a lymphoid follicle in the white pulp. (L) Plot representing number of granulomas (necrotizing and nonnecrotizing) counted in the spleen sections from treated and untreated animals. The blue lines represent the average values for the untreated or treated sets of animals. (M) Splenomegaly was observed in infected guinea pigs that did not receive AZD0530 treatment (bar, 1 cm). G, granuloma; N, necrosis; E, epithelioid cells; AS, alveolar space; RP, red pulp; WP, white pulp; FC, follicular center. The images depicted here are stained with hematoxylin and eosin. (N) Phosphorylation of IκBα in H37Rv-infected THP-1 macrophages that were either untreated or treated with AZD0530 (20 µM) at 48 h postinfection.

Lung and spleen tissues harvested from the euthanized animals were subjected to histopathological analyses. We observed several granulomatous lesions in the lung sections from untreated animals (Fig. 4A). At low magnification, ×40, the presence of multiple granulomas in the lung parenchyma can be easily visualized (Fig. 4B). Many of the granuloma lesions from untreated animals, when visualized at higher magnification (×100), showed central necrotic core, a sign of excessive inflammation and active disease (Fig. 4C). Interestingly, lung sections from AZD0530-treated animals showed very few granuloma lesions and none of those lesions had necrotic core (Fig. 4D to F). Numbers of granulomas (necrotizing or nonnecrotizing) scored in the lungs of treated or untreated animals are summarized in Fig. 4G. We also calculated total granuloma scores, which assume different weights for necrotizing (5 each) and nonnecrotizing (2.5 each) granulomas as well as percent organ area occupied by the granuloma in each case. The data showing this calculation for lung sections from treated and untreated animals are presented in Table 2, clearly suggesting effective control of disease pathology in the treated animals. A better prognosis for TB infection in the drug-treated animals was further confirmed from the histopathological analysis of spleen tissues. Spleens from the infected animals that were not administered the drug showed massive inflammation and were full of granulomatous lesions, which were necrotic at the core (Fig. 4H and I). Necrotic granulomas were missing from the spleens of AZD0530-treated infected animals in the sections analyzed in this study (Fig. 4J and K). Numbers of granulomas (necrotizing or nonnecrotizing) scored in the spleen of treated or untreated animals are summarized in Fig. 4L. As can be inferred from Fig. 4L, the spleen of all of the animals, except one, that received chemotherapy showed no granulomatous lesions in the sections analyzed in this study. We also calculated the granuloma score and percent organ area occupied by the granuloma for spleens as was done for the lungs, clearly showing control of pathology by AZD0530 treatment (Table 3). In addition to the above, we also measured the size of spleens isolated from the test animals. As shown in Fig. 4M, splenomegaly was significant in the untreated animals (median size, 4.5 cm; minimum, 3.5 cm; maximum, 6.5 cm) while the treated animals showed normal spleen size (median size, 3 cm; minimum, 2 cm; maximum, 3 cm [Fig. 4M]). Therefore, inhibition of Src tyrosine kinase, by AZD0530, not only resulted in *M. tuberculosis* killing but also led to alleviation of the classical pathological symptoms of TB infection. Incidentally, in THP-1 macrophages, treatment with AZD0530 resulted in decreased phosphorylation of IκBα (Ser32) (Fig. 4N). Phosphorylation of IκBα signals its subsequent proteosomal degradation, resulting in translocation of NF-κB to the nucleus, where it regulates the expression of genes belonging to the inflammatory pathways (23), suggesting inhibition of NF-κB activation as a possible mechanism for the control of inflammation by this drug.

**TABLE 2:** Granuloma score for lung samples

Lung sample	No. of granulomas (score)			Total granuloma		% of organ area occupied by granuloma
	With necrosis (5)	With no necrosis (2.5)	With fibrosis (1)	Score	Count	
Treated						
1	0	3.0	0	7.5	3	<5
2	0	4.0	0	10	4	<5
3	0	10.0	0	25	10	20
4	0	11.0	0	27.5	11	30
5	0	18.0	0	45	18	25
Control						
1	3	14.0	0	50	17	40
2	6	24.0	0	90	30	50
3	0	12.0	0	30	12	30
4	3	15.0	0	52.5	18	30
5	0	20.0	0	50	20	40

**TABLE 3:** Granuloma score for spleen samples

Spleen sample	No. of granulomas (score)			Total granuloma		% of organ area occupied by granuloma
	With necrosis (5)	With no necrosis (2.5)	With fibrosis (1)	Score	Count	
Treated						
1	0	0	0	0	0	0
2	0	0	0	0	0	0
3	0	0	0	0	0	0
4	0	11.0	0	7.5	11	5
5	0	0	0	0	0	0
Control						
1	0	4.0	0	10	4	5
2	3	7.0	0	32.5	10	50
3	6	24.0	0	90	30	60
4	10	24.0	0	110	34	75
5	0	>30	0	>75	>30	45

## DISCUSSION

Host-directed approaches to target tuberculosis have gained momentum in the recent past owing to several studies that identified crucial roles played by select host molecules in the intramacrophage survival of *M. tuberculosis* (11, 17, 24, 25). We reported earlier that Src tyrosine kinase activity was required for survival of virulent *M. tuberculosis* through regulating phagosome maturation and autophagy induction in macrophages (13). In this report, we show that by inhibiting Src kinases using AZD0530, we achieved a considerable reduction in the intracellular pathogen load in H37Rv-infected THP-1 macrophages. The concentration of AZD0530 used in the study was much higher than the known 50% inhibitory concentration (IC_50_) of this molecule against purified Src kinases (~3 nM). However, in THP-1 macrophages, we could achieve significant Src inhibition only at concentrations higher than 10 µM. In a few cell-based assays in the past, higher doses of AZD0530 were used (14). It was shown that AZD0530 could inhibit cellular migration speed by about 50% and ~80% at 1 µM and 10 µM, respectively (14). Interestingly, they show about 80 and 90% reduction in the phosphorylation of paxillin (Y118) at 10 and 25 µM AZD0530, respectively (14). The dose response of paxillin phosphorylation in that study was similar to the dose response of Src phosphorylation in our study. Paxillin, as is known, gets phosphorylated by focal adhesion kinase (FAK), which in turn is regulated through phosphorylation by Src (26, 27). Together, there seems a possibility that in *in vitro* studies, the required concentration of AZD0530 to effectively inhibit Src is much higher. To note, interference in drug activity due to the presence of serum in cell culture studies is well known (28). This could, in principle, explain the higher concentration of AZD0530 required for getting the desired effects in this study. Our results revealed that even drug-resistant clinical isolates of *M. tuberculosis*, namely, JAL2261, 1934, and MYC431, were sensitive to AZD0530 treatment. The drug AZD0530 is a dual Src/Abl inhibitor and is known to inhibit Abl kinase, albeit at much lower efficiency. Our Western blot data showed no significant inhibition in Abl phosphorylation upon AZD0530 treatment at the tested doses, suggesting that the effects studied were mostly Src mediated. However, *in vivo* we could not establish if Abl activity was also inhibited and therefore cannot rule out that some of the effects of AZD0530 treatment on bacterial survival in the guinea pigs may also be due to Abl inhibition. It is especially important since inhibition of Abl kinase independently has been shown to control mycobacterial survival (11).

Mechanistic insights into the mode of AZD0530 action revealed that drug treatment enhanced the host antimycobacterial processes of phagosomal acidification and autophagy. We observed higher lysosomal acidification and localization of *M. tuberculosis* to acidified lysosomes in cells treated with Src inhibitor, which was expected, as shown previously (13). The exact mechanism through which Src influences phagosomal acidification remains unclear. Interestingly, another host tyrosine kinase, Abl, has also been shown to modulate phagosomal acidification by influencing transcription of a proton-pumping enzyme, vacuolar-type H^+^-ATPase (12). It has been reported that inhibition of Abl activity leads to enhanced phagosomal acidification (11). In addition, AZD0530 treatment blocked signaling through the Src/phosphatidylinositol 3-kinase (PI3K)/AKT axis, which influences many downstream signaling pathways (29, 30). One of the key molecules downstream of AKT is mToR, which is a negative regulator of autophagy (19, 20). Our results show that in AZD0530-treated infected macrophages, xenophagy flux of *M. tuberculosis*-containing autophagosomes increases, which could potentially explain the reduced *M. tuberculosis* survival upon treatment. The role of selective xenophagy flux in regulating intracellular survival of *M. tuberculosis* has been shown recently (18).

In animal models, use of host factor inhibitors to successfully check *M. tuberculosis* survival has been reported in very few studies (11, 24). Therefore, it was encouraging to observe high efficacy of AZD0530 against H37Rv as well as the XDR strain MYC431. The chemotherapy with AZD0530 also inhibited hematogenous dissemination of the pathogen in guinea pigs, leading to a significantly decreased (up to ~10-fold) bacillary load in the spleen. Since the host-directed approaches are expected to provide opportunity to target both drug-sensitive and -resistant strains using the same drug, results here with the XDR strain provide proof of concept for feasibility of such approaches in the future.

Histological examination of the lung and spleen showed that animals that received chemotherapy had significantly decreased granulomatous lesions as well as necrotic granulomas in the lung and spleen tissues. Moreover, splenomegaly was dramatically reduced in the treated animals. Infection of macrophages with *M. tuberculosis* induces the production of proinflammatory cytokines such as tumor necrosis factor (TNF), gamma interferon (IFN-γ), etc. (31). Although important to control *M. tuberculosis* infection, an excessive inflammatory response can lead to tissue damage and lung injury. The dual effect of TNF in mediating resistance as well as susceptibility in mycobacterial infections has been elucidated recently (32). Therefore, in addition to killing the pathogen, managing inflammation is an important part of TB treatment. NF-κB is a transcription factor that regulates vital processes such as inflammation. TNF-α is a major proinflammatory cytokine that relays its downstream effects via activation of NF-κB. In a latent form, NF-κB resides in the cytoplasm as a complex with its inhibitory protein IκB. Upon stimulation, IκB is phosphorylated and targeted for degradation, thereby releasing NF-κB. Once free, NF-κB translocates to the nucleus, where it regulates transcription of various genes from the inflammatory pathways (23). Src family kinases (SFKs) are reported to influence cytokine signaling and inflammatory responses (33). Several studies have demonstrated the role of SFKs in LPS-induced production of TNF-α and other inflammatory cytokines (34, 35). The use of PP2, a selective inhibitor of SFKs, in mouse models has indeed proved effective in reducing lipopolysaccharide (LPS)-induced lung injury (36). NF-κB activation is regulated by Src in macrophages and epithelial cells stimulated with TNF-α, LPS, or hypoxia (37). We observed a reduction in phosphorylation of IκBα upon AZD0530 treatment in H37Rv-infected THP-1 macrophages. This suggests that AZD0530 treatment could control the NF-κB-mediated proinflammatory signaling in *M. tuberculosis*-infected animals. Interestingly, animals lacking ATG5, a key autophagy regulatory molecule, show massive inflammation and lung pathology upon infection with *M. tuberculosis* (38). In agreement with this, recently we have also shown that autophagy inhibition was correlated with increased inflammatory phenotype in the activated macrophages (39). Here, we show that Src inhibition by AZD0530 leads to higher flux of *M. tuberculosis*-containing autophagosomes. Thus, controlled inflammation in AZD0530-treated animals could also be due to heightened autophagy response in these animals.

In conclusion, inhibition of the Src tyrosine kinases proved to be effective against both drug-sensitive and -resistant strains of *M. tuberculosis.* AZD0530 successfully reduced pathogen loads both *in vitro* and *in vivo.* Additionally, drug treatment alleviated disease-associated pathology in guinea pigs. While this study provides proof of concept for the use of Src kinase inhibitors as drugs against tuberculosis, further examination is warranted to determine the optimal dosages and treatment regimens, including drug combinations.

## MATERIALS AND METHODS

### Ethics statement.

All the experiments on animals were approved by the Institutional Animal Ethics Committee (IAEC), approval no. ICGEB/AH/2012/02/IMM-31.

### Cell culture.

The human promonocytic cell line THP-1 (American Type Culture Collection) was cultured in RPMI 1640 (Gibco Laboratories) medium supplemented with 10% fetal calf serum (FCS) (HyClone) and was maintained at 37°C in a humidified, 5% CO_2_ atmosphere. THP-1 cells were differentiated using 20 ng/ml of phorbol myristate acetate (PMA; Sigma) for a minimum of 20 h.

### Bacterial culture.

Mycobacteria were cultured in Middlebrook 7H9 broth (Difco) supplemented with 10% albumin-dextrose-catalase (ADC) (Becton, Dickinson), 0.4% glycerol, and 0.05% Tween 80 until the mid-log phase. Bacterial strains used in this study were available in the Tuberculosis Aerosol Challenge Facility (TACF) at the International Centre for Genetic Engineering and Biotechnology (ICGEB).

### *In vitro Mycobacterium tuberculosis* infections.

Single-cell bacterial suspensions were prepared by aspiration five times each with a 23- and then a 26-gauge needle, followed by an additional dispersion for 3 times through a 30-gauge needle. Quantitation of bacteria was done by measuring absorbance at a 600-nm wavelength (0.6 optical density [OD] value corresponds to ~100 × 10^6^ bacteria). Complete medium containing the required number of bacteria was added to the PMA-differentiated THP-1 cells at an MOI of 10. After 4 h of infection, the cells were washed twice with warm RPMI medium and treated with amikacin (200 µg/ml) to remove and kill any extracellular bacteria. After 2 h of amikacin treatment, complete medium was added to the cells. This was referred to as the 0-h time point in the experiments, i.e., 4 + 2 h post-*M. tuberculosis* addition.

### CFU assay.

The infected cells were lysed in 7H9 medium containing 0.06% SDS. Serial dilutions of this lysate were plated on 7H11 agar (Difco) containing square petri plates supplemented with 10% oleic acid-albumin-dextrose-catalase (OADC) (Becton, Dickinson) and 0.5% glycerol. On one side of the square plate, 10 µl of the cell lysate was spotted and the plate was tilted to allow the sample to gently flow along the surface in parallel tracks. The plates were incubated at 37°C to allow bacterial growth, and counts were performed after 14 and 21 days.

### MTT cell viability assay.

Cell viability was assessed by the MTT [3-(4,5-dimethyl-2-thiazolyl)-2,5-diphenyl-2H-tetrazolium bromide] assay. MTT (Sigma) was added to THP-1 macrophages at a final working concentration of 1 mg/ml. The cells were incubated for 1 h at 37°C and a 5% CO_2_ atmosphere. This was followed by addition of 100 µl dimethyl sulfoxide (DMSO) to the samples to dissolve the formazan crystals. Quantitation was done by measuring sample absorbance at a 560-nm wavelength.

### alamarBlue cell viability assay.

The alamarBlue microplate assay was performed with 5 × 10^5^ bacilli per ml seeded in 7H9 medium supplemented with the indicated inhibitors. The bacterial cultures were incubated for 7 days. At the end of the treatment period, addition of alamarBlue was done at 10% of the total culture volume. Fluorescence measurements were made after a 24-h incubation at 530-nm/590-nm (excitation/emission) wavelengths.

### Guinea pig aerosol challenge.

All animal experiments were carried out in the Tuberculosis Aerosol Challenge Facility (TACF, ICGEB, New Delhi, India). Outbred Dunkin-Hartley guinea pigs (National Institute of Nutrition, Hyderabad, India) were housed in cages contained within a biosafety level 3 laminar flow enclosure and were weighed once every 2 weeks. Aerosol challenge was given to the animals in a Wisconsin-Madison chamber according to the protocol standardized in-house to deliver a high dose of ~100 bacilli/lung. To check for infection establishment, four animals were selected randomly and humanely euthanized 15 days post-aerosol challenge. The lungs and spleen tissues were aseptically harvested. The weights of the whole organs and the portions used for homogenization were measured. For the estimation of bacterial CFU, the right cranial lobe of lung and a portion of the spleen were homogenized. Tissue lysates were serially diluted and plated on petri plates containing Middlebrook 7H11 agar (Difco) supplemented with 10% OADC (Becton, Dickinson) and 0.5% glycerol.

### AZD0530 treatment.

For *in vitro* experiments, AZD0530 (Reagents Direct) was added to the culture medium at final working concentrations of 20 µM and 40 µM at the 0-h time point. For *in vivo* experiments, drug dosing was initiated 15 days post-aerosol challenge and the animals were administered the drug by oral gavage at a dose of 10 mg/kg of body weight every alternate day. Our study tested AZD0530 inhibitor for the first time in a guinea pig TB model, and therefore, we had no references to determine the doses to be administered. In order to address this, we converted the AZD0530 dose tested previously in humans and calculated the human-equivalent dose in guinea pigs according to the FDA guidelines. We also referenced the doses used in mouse studies (40).

### Histological examination.

Following euthanasia, portions of the lung and spleen tissues were infused with 10% neutral buffered formalin and preserved until processed for histopathological assessment. At the time of processing, the tissues were embedded in paraffin and stained with hematoxylin and eosin (HE) for histologic evaluation and imaging. Granuloma scoring was performed by observing and manually counting individual granulomas with the appropriate magnification. Granulomas with necrosis were given a score of 5, granulomas with no necrosis were given a score of 2.5, and granulomas with fibrosis were given a score of 1. These scores were summed up to give the total granuloma score for a particular sample. The percentage of organ area occupied was calculated as the ratio of total area of the granuloma to the total area of the whole section.

### Confocal microscopy.

The required number of bacteria were stained with PKH67 (Sigma), a green lipophilic dye, and were used for infecting PMA-differentiated THP-1 cells. To visualize acidified lysosomes, LysoTracker red dye (LysoTracker Red DND-99; Life Technologies) was added to the sample wells at a concentration of 200 nM for 1 h. The cells were then fixed in 4% paraformaldehyde and mounted using ProLong Gold antifade reagent (Life Technologies). Images were acquired by NIS-Elements software using the Nikon A1R laser scanning confocal microscope equipped with a Nikon Plan Apo 60× 1.40-numerical-aperture (NA) oil immersion objective. Serial confocal sections, 0.5 µm thick, were acquired within a *z*-stack spanning 10 to 15 µm. For a particular image, the *z*-sections were collapsed into a single image. Percentage of *M. tuberculosis* bacteria (green) colocalizing with lysosomes (red) was determined from the collapsed composite images. Immunostaining was performed according to the protocol mentioned elsewhere (18). Anti-LC3 antibody was purchased from Cell Signaling Technologies. Colocalization coefficient (M1) was calculated as discussed earlier (18). LysoTracker intensity was measured using ImageJ software with the following formula: [integrated density − (area of selected cell × mean fluorescence intensity of background)].

### NMR spectroscopy.

NMR samples were prepared by dissolving 100 µg commercially obtained AZD0530 in 180 µl CDCl_3_ (99.8% D) containing 1% (vol/vol) tetramethylsilane (TMS) from Aldrich (catalog no. 151831) per the protocol described in the work of Beckonert et al. (41). One-dimensional (1D) ^1^H NMR spectra using the Carr-Purcell-Meiboom-Gill (CPMG) pulse scheme (to enhance the signals from the small molecule) were measured at 25°C on a Bruker Avance III spectrometer equipped with cryogenic triple-resonance 5-mm TCI probe head, operating at the field strength of 500.13 MHz. Acquisition times and spectral widths for ^1^H dimension were 4.09 s (*t*_max_) and 8,012.82 Hz, respectively. The number of scans used for AZD0530 was 32. Topspin 2.1 (Bruker AG) software was used for acquisition, Fourier transformation, and processing of time-domain data. ^1^H chemical shifts referenced directly the TMS methyl proton resonance at 0 ppm in all spectra.

### Statistical analyses.

Statistical significance of results obtained through experimentation was determined using Student’s two-tailed *t* test. For the animal experiments, *P* values were determined using the Mann-Whitney U rank sum test.

## References

[1] O’Brien RJ, Nunn PP 2001 The need for new drugs against tuberculosis. Obstacles, opportunities and next steps. Am J Crit Care Med 162:1055–1058. 10.1164/ajrccm.163.5.2007122 11316634

[2] Kellam P 2006 Attacking pathogens through their hosts. Genome Biol 7:201. doi:10.1186/gb-2006-7-1-201. 16515720 PMC1431708

[3] Burton EA, Plattner R, Pendergast AM 2003 Abl tyrosine kinases are required for infection by Shigella flexneri. EMBO J 22:5471–5479. doi:10.1093/emboj/cdg512. 14532119 PMC213767

[4] Ly KT, Casanova JE 2009 Abelson tyrosine kinase facilitates *Salmonella enterica* serovar Typhimurium entry into epithelial cells. Infect Immun 77:60–69. doi:10.1128/IAI.00639-08. 18936177 PMC2612273

[5] Pielage JF, Powell KR, Kalman D, Engel JN 2008 RNAi screen reveals an Abl kinase-dependent host cell pathway involved in Pseudomonas aeruginosa internalization. PLoS Pathog 4:e1000031. doi:10.1371/journal.ppat.1000031. 18369477 PMC2265438

[6] Tammer I, Brandt S, Hartig R, König W, Backert S 2007 Activation of Abl by Helicobacter pylori: a novel kinase for CagA and crucial mediator of host cell scattering. Gastroenterology 132:1309–1319. doi:10.1053/j.gastro.2007.01.050. 17408661

[7] Reeves PM, Bommarius B, Lebeis S, McNulty S, Christensen J, Swimm A, Chahroudi A, Chavan R, Feinberg MB, Veach D, Bornmann W, Sherman M, Kalman D 2005 Disabling poxvirus pathogenesis by inhibition of Abl-family tyrosine kinases. Nat Med 11:731–739. doi:10.1038/nm1265. 15980865

[8] Swimm A, Bommarius B, Li Y, Cheng D, Reeves P, Sherman M, Veach D, Bornmann W, Kalman D 2004 Enteropathogenic Escherichia coli use redundant tyrosine kinases to form actin pedestals. Mol Biol Cell 15:3520–3529. doi:10.1091/mbc.E04-02-0093. 15155808 PMC491815

[9] Lechartier B, Rybniker J, Zumla A, Cole ST 2014 Tuberculosis drug discovery in the post-post-genomic era. EMBO Mol Med 6:158–168. doi:10.1002/emmm.201201772. 24401837 PMC3927952

[10] Stanley SA, Barczak AK, Silvis MR, Luo SS, Sogi K, Vokes M, Bray MA, Carpenter AE, Moore CB, Siddiqi N, Rubin EJ, Hung DT 2014 Identification of host-targeted small molecules that restrict intracellular *Mycobacterium tuberculosis* growth. PLoS Pathog 10:e1003946. doi:10.1371/journal.ppat.1003946. 24586159 PMC3930586

[11] Napier RJ, Rafi W, Cheruvu M, Powell KR, Zaunbrecher MA, Bornmann W, Salgame P, Shinnick TM, Kalman D 2011 Imatinib-sensitive tyrosine kinases regulate mycobacterial pathogenesis and represent therapeutic targets against tuberculosis. Cell Host Microbe 10:475–485. doi:10.1016/j.chom.2011.09.010. 22100163 PMC3222875

[12] Bruns H, Stegelmann F, Fabri M, Döhner K, van Zandbergen G, Wagner M, Skinner M, Modlin RL, Stenger S 2012 Abelson tyrosine kinase controls phagosomal acidification required for killing of Mycobacterium tuberculosis in human macrophages. J Immunol 189:4069–4078. doi:10.4049/jimmunol.1201538. 22988030 PMC3684563

[13] Karim AF, Chandra P, Chopra A, Siddiqui Z, Bhaskar A, Singh A, Kumar D 2011 Express path analysis identifies a tyrosine kinase Src-centric network regulating divergent host responses to Mycobacterium tuberculosis infection. J Biol Chem 286:40307–40319. doi:10.1074/jbc.M111.266239. 21953458 PMC3220550

[14] Green TP, Fennell M, Whittaker R, Curwen J, Jacobs V, Allen J, Logie A, Hargreaves J, Hickinson DM, Wilkinson RW, Elvin P, Boyer B, Carragher N, Plé PA, Bermingham A, Holdgate GA, Ward WH, Hennequin LF, Davies BR, Costello GF 2009 Preclinical anticancer activity of the potent, oral Src inhibitor AZD0530. Mol Oncol 3:248–261. doi:10.1016/j.molonc.2009.01.002. 19393585 PMC5527863

[15] Hennequin LF, Allen J, Breed J, Curwen J, Fennell M, Green TP, Lambert-van der Brempt C, Morgentin R, Norman RA, Olivier A, Otterbein L, Plé PA, Warin N, Costello G 2006 *N*-(5-Chloro-1,3-benzodioxol-4-yl)-7-[2-(4-methylpiperazin-1-yl)ethoxy]-5-(tetrahydro-2H-pyran-4-yloxy)quinazolin-4-amine, a novel, highly selective, orally available, dual-specific c-Src/Abl kinase inhibitor. J Med Chem 49:6465–6488. doi:10.1021/jm060434q. 17064066

[16] Armstrong JA, Hart PD 1971 Response of cultured macrophages to Mycobacterium tuberculosis, with observations on fusion of lysosomes with phagosomes. J Exp Med 134:713–740. doi:10.1084/jem.134.3.713. 15776571 PMC2139093

[17] Kumar D, Nath L, Kamal MA, Varshney A, Jain A, Singh S, Rao KV 2010 Genome-wide analysis of the host intracellular network that regulates survival of Mycobacterium tuberculosis. Cell 140:731–743. doi:10.1016/j.cell.2010.02.012. 20211141

[18] Chandra P, Ghanwat S, Matta SK, Yadav SS, Mehta M, Siddiqui Z, Singh A, Kumar D 2015 Mycobacterium tuberculosis inhibits RAB7 recruitment to selectively modulate autophagy flux in macrophages. Sci Rep 5:16320. doi:10.1038/srep16320. 26541268 PMC4635374

[19] Kroemer G, Mariño G, Levine B 2010 Autophagy and the integrated stress response. Mol Cell 40:280–293. doi:10.1016/j.molcel.2010.09.023. 20965422 PMC3127250

[20] Memmott RM, Dennis PA 2009 Akt-dependent and -independent mechanisms of mTOR regulation in cancer. Cell Signal 21:656–664. doi:10.1016/j.cellsig.2009.01.004. 19166931 PMC2650010

[21] Ahmad Z, Klinkenberg LG, Pinn ML, Fraig MM, Peloquin CA, Bishai WR, Nuermberger EL, Grosset JH, Karakousis PC 2009 Biphasic kill curve of isoniazid reveals the presence of drug-tolerant, not drug-resistant, Mycobacterium tuberculosis in the guinea pig. J Infect Dis 200:1136–1143. doi:10.1086/605605. 19686043

[22] Baselga J, Cervantes A, Martinelli E, Chirivella I, Hoekman K, Hurwitz HI, Jodrell DI, Hamberg P, Casado E, Elvin P, Swaisland A, Iacona R, Tabernero J 2010 Phase I safety, pharmacokinetics, and inhibition of SRC activity study of saracatinib in patients with solid tumors. Clin Cancer Res 16:4876–4883. doi:10.1158/1078-0432.CCR-10-0748. 20805299

[23] Hayden MS, West AP, Ghosh S 2006 NF-kappaB and the immune response. Oncogene 25:6758–6780. doi:10.1038/sj.onc.1209943. 17072327

[24] Jayaswal S, Kamal MA, Dua R, Gupta S, Majumdar T, Das G, Kumar D, Rao KV 2010 Identification of host-dependent survival factors for intracellular Mycobacterium tuberculosis through an siRNA screen. PLoS Pathog 6:e1000839. doi:10.1371/journal.ppat.1000839. 20419122 PMC2855445

[25] Sundaramurthy V, Barsacchi R, Samusik N, Marsico G, Gilleron J, Kalaidzidis I, Meyenhofer F, Bickle M, Kalaidzidis Y, Zerial M 2013 Integration of chemical and RNAi multiparametric profiles identifies triggers of intracellular mycobacterial killing. Cell Host Microbe 13:129–142. doi:10.1016/j.chom.2013.01.008. 23414754

[26] Bellis SL, Miller JT, Turner CE 1995 Characterization of tyrosine phosphorylation of paxillin in vitro by focal adhesion kinase. J Biol Chem 270:17437–17441. doi:10.1074/jbc.270.29.17437. 7615549

[27] Calalb MB, Polte TR, Hanks SK 1995 Tyrosine phosphorylation of focal adhesion kinase at sites in the catalytic domain regulates kinase activity: a role for Src family kinases. Mol Cell Biol 15:954–963. doi:10.1128/MCB.15.2.954. 7529876 PMC231984

[28] Kratochwil NA, Huber W, Müller F, Kansy M, Gerber PR 2002 Predicting plasma protein binding of drugs: a new approach. Biochem Pharmacol 64:1355–1374. doi:10.1016/S0006-2952(02)01074-2. 12392818

[29] Datta K, Bellacosa A, Chan TO, Tsichlis PN 1996 Akt is a direct target of the phosphatidylinositol 3-kinase. Activation by growth factors, v-src and v-Ha-ras, in Sf9 and mammalian cells. J Biol Chem 271:30835–30839. doi:10.1074/jbc.271.48.30835. 8940066

[30] Haynes MP, Li L, Sinha D, Russell KS, Hisamoto K, Baron R, Collinge M, Sessa WC, Bender JR 2003 Src kinase mediates phosphatidylinositol 3-kinase/Akt-dependent rapid endothelial nitric-oxide synthase activation by estrogen. J Biol Chem 278:2118–2123. doi:10.1074/jbc.M210828200. 12431978

[31] Flynn JL, Chan J 2001 Immunology of tuberculosis. Annu Rev Immunol 19:93–129. doi:10.1146/annurev.immunol.19.1.93. 11244032

[32] Roca FJ, Ramakrishnan L 2013 TNF dually mediates resistance and susceptibility to mycobacteria via mitochondrial reactive oxygen species. Cell 153:521–534. doi:10.1016/j.cell.2013.03.022. 23582643 PMC3790588

[33] Page TH, Smolinska M, Gillespie J, Urbaniak AM, Foxwell BM 2009 Tyrosine kinases and inflammatory signalling. Curr Mol Med 9:69–85. doi:10.2174/156652409787314507. 19199943

[34] Fan H, Teti G, Ashton S, Guyton K, Tempel GE, Halushka PV, Cook JA 2003 Involvement of Gi proteins and Src tyrosine kinase in TNFα production induced by lipopolysaccharide, group B streptococci and Staphylococcus aureus. Cytokine 22:126–133. doi:10.1016/S1043-4666(03)00122-4. 12842760

[35] Smolinska MJ, Horwood NJ, Page TH, Smallie T, Foxwell BM 2008 Chemical inhibition of Src family kinases affects major LPS-activated pathways in primary human macrophages. Mol Immunol 45:990–1000. doi:10.1016/j.molimm.2007.07.026. 17875324

[36] Severgnini M, Takahashi S, Tu P, Perides G, Homer RJ, Jhung JW, Bhavsar D, Cochran BH, Simon AR 2005 Inhibition of the Src and Jak kinases protects against lipopolysaccharide-induced acute lung injury. Am J Respir Crit Care Med 171:858–867. doi:10.1164/rccm.200407-981OC. 15665321

[37] Oeckinghaus A, Hayden MS, Ghosh S 2011 Crosstalk in NF-kappaB signaling pathways. Nat Immunol 12:695–708. doi:10.1038/ni.2065. 21772278

[38] Castillo EF, Dekonenko A, Arko-Mensah J, Mandell MA, Dupont SJ, Delgado-Vargas M, Timmins GS, Bhattacharya D, Yang H, Hutt J, Lyons CR, Dobos KM, Deretic V. 2012 Autophagy protects against active tuberculosis by suppressing bacterial burden and inflammation. Proc Natl Acad Sci U S A 109:E3168–E3176. doi:10.1073/pnas.1210500109. 23093667 PMC3503152

[39] Matta SK, Kumar D 2015 AKT mediated glycolytic shift regulates autophagy in classically activated macrophages. Int J Biochem Cell Biol 66:121–133. doi:10.1016/j.biocel.2015.07.010. 26222186

[40] Chang YM, Bai L, Liu S, Yang JC, Kung HJ, Evans CP 2008 Src family kinase oncogenic potential and pathways in prostate cancer as revealed by AZD0530. Oncogene 27:6365–6375. doi:10.1038/onc.2008.250. 18679417 PMC4294546

[41] Beckonert O, Keun HC, Ebbels TM, Bundy J, Holmes E, Lindon JC, Nicholson JK 2007 Metabolic profiling, metabolomic and metabonomic procedures for NMR spectroscopy of urine, plasma, serum and tissue extracts. Nat Protoc 2:2692–2703. doi:10.1038/nprot.2007.376. 18007604

